# High Sucrose and Cholic Acid Diet Triggers PCOS-like Phenotype and Reduces Enterobacteriaceae Colonies in Female Wistar Rats

**DOI:** 10.3390/pathophysiology29030026

**Published:** 2022-07-08

**Authors:** I Made Putra Juliawan, Febie Putra Suwana, Jimmy Yanuar Annas, Muhammad Firman Akbar, Widjiati Widjiati

**Affiliations:** 1 Department of Obstetrics and Gynecology, Faculty of Medicine, Airlangga University, Surabaya 75320, Indonesia; putrajuliawan75@gmail.com (I.M.P.J.); jimmyyanuar@gmail.com (J.Y.A.); 2 Department of Obstetrics and Gynecology, Faculty of Medicine, The University of Mataram, Mataram 83115, Indonesia; 3 Department of Obstetrics and Gynecology, General Hospital of West Nusa Tenggara, Mataram 83127, Indonesia; febieputrasuwana@gmail.com; 4 Graduate Institute of Biomedical Sciences, College of Medicine, China Medical University, Taichung 400, Taiwan; 5 Centre of Medical Education and Research, The University of Mataram Teaching Hospital, Mataram 83115, Indonesia; 6 Department of Veterinary Anatomy, Faculty of Veterinary Medicine, Airlangga University, Surabaya 75320, Indonesia

**Keywords:** polycystic ovary syndrome, insulin resistance, hyperandrogenism, cholic acid, sucrose-rich diet, *Enterobacteriaceae*

## Abstract

Polycystic ovary syndrome (PCOS), a common hormonal disorder in women of reproductive age, is associated with a poor and unhealthy diet. This study aimed to investigate the effect of a high sucrose and cholic acid (HSCA) diet in the presence of PCOS-like phenotypes. Female Wistar rats were divided into HSCA and normal diet groups for four weeks, each with twenty rats. Body weight was assessed before and after the study. Blood and fecal samples were obtained to measure HOMA-IR and testosterone level (ELISA) and *Enterobacteriaceae* isolates grown on MacConkey Agar. Obtained ovarian tissues were H&E-stained. HSCA rats demonstrated a reduction in *Enterobacteriaceae* colonies (median 4.75 × 10^5^ vs. 2.47 × 10^4^/CFU, *p* < 0.001) and an elevated HOMA-IR (mean 2.94 ± 1.30 vs. 4.92 ± 0.51, *p* < 0.001), as well as an increase in testosterone level (median 0.65 vs. 3.00 ng/mL, *p* < 0.001), despite no statistical differences in the change in body weight (mean −2.31 ± 14.42 vs. −3.45 ± 9.32, *p* = 0.769). In H&E staining, HSCA rats had a reduction in preovulatory follicle count (median 0.50 vs. 0.00, *p* = 0.005). The HSCA diet caused insulin resistance and high testosterone levels, which contribute to the development of PCOS, and affected folliculogenesis by altering follicular maturation, but had no effect on ovulation.

## 1. Introduction

Polycystic ovary syndrome (PCOS), described as the presence of polycystic ovaries, ovulatory dysfunction, and hyperandrogenism, is one of the most common hormonal disorders in premenopausal women and leads to a decrease in quality of life [1,2,3]. The worldwide prevalence of PCOS accounts for 1.6–4% among reproductive age, but the estimate increases to approximately 18–20% due to the complexity of diagnostic criteria and phenotypes [4,5,6].

Although the Rotterdam consensus has well-described the PCOS criteria and has been used extensively for diagnosis, the phenotype is heterogeneous and cannot be captured by the established definition [7]. Clinical signs are not limited to only anovulation, polycystic ovarian, and an increasing level of testosterone, but also present an increase in insulin resistance and body weight, as well as an alteration of gut microbiota composition [8]. The etiology of PCOS is still unknown. However, Rosenfield and Ehrmann [9] describe that there are many predisposing factors to PCOS, such as genetic and environmental factors. Dietary intake is one of the important environmental factors that cause PCOS [10]. A case-controlled study by Barrea et al. [11] showed that a high-fiber and monounsaturated fat diet can be a supportive therapy to improve insulin resistance and hyperandrogenism in pathogenesis. Additionally, diet is a key factor that can modify the composition and stability of the enteric microbiota, which could eventually contribute to the development of PCOS [12]. This evidence indicates that the diet can indirectly or directly induce PCOS by triggering gut microbiota dysbiosis.

An observational study suggested that sucrose beverages have been positively correlated with the prevalence of PCOS among Brazilians [13]. This finding also showed that a high-sucrose diet induces increased blood glucose, reducing insulin sensitivity, and promotes fat tissue accumulation in vivo [13]. Despite a clear correlation between high-sucrose intake and PCOS [13], less is known about the effect of a combination of high-sucrose and high-cholic acid (HSCA) dietary intake. Cholic acid, a primary water-insoluble bile acid, is thought to have a link with PCOS. Studies have shown that cholic acid is elevated in PCOS women [14] and there is a positive correlation between hyperandrogenism and conjugated primary bile acid levels [15]. Furthermore, cholic acid levels in the gut may have an association with the composition of the fecal microbiota in individuals with PCOS [16]. The *Enterobacteriaceae* family is found to be elevated in patients with PCOS and may play an important role in the pathophysiology [17]. Therefore, the objective of the present in vivo study was to evaluate the impact of the HSCA diet on the *Enterobacteriaceae*, insulin sensitivity, testosterone level, and histological parameters in female *Rattus novergicus* ovaries. In this study, PCOS features were not all observed under HSCA treatment. However, our findings showed that HSCA promoted insulin resistance, increased testosterone levels, and reduced *Enterobacteriaceae* colonies. The present study also found that HOMA-IR (Homeostatic Model Assessment for Insulin Resistance) has a positive association with testosterone level and the antral follicle count is correlated with corpus luteum. In addition, this study showed that HSCA leads to deterioration in follicle maturation, but ovulation remains presented.

## 2. Materials and Methods

- Animals

Female Wistar rats of 3 months old and with a body weight of 160–180 g were supplied by the Faculty of Veterinary Medicine, University of Airlangga, and kept in the Animal Unit under ambient air humidity (60–70%) and temperature (21–25 °C). Rats were provided ad libitum access to food and water. All procedures were carried out according to and approved by the Animal Care and Use Committee (ACUC), Faculty of Veterinary Medicine, Airlangga University, Surabaya, Indonesia (30 July 2021, No.: 2.KE.098.07.2021).

b. Study Design

Forty Wistar rats were equally randomized into two groups, one group receiving normal food (Envigo–LM-485, 3.1 kcal/g, 17% calorie from fat) and the other fed with a high-sucrose (HS) diet (32% glucose). The high-sucrose diet group was supplemented with 40% cholic acid (CA) by using a probe (CAS: 81-25-5, product number C0324). The duration of food treatment was 4 weeks. The study was a randomized, controlled, post-test only measurement design. Before randomization, all rats were measured for body weight and left for 7 days for adaptation. A vaginal examination was performed to determine the estrous cycle, an analogue of the human menstrual cycle, which has four phases, namely proestrus, estrus, metestrus, and diestrus [18]. The purpose of this examination is to ensure that the rats are within the estrus phase, which is related to ovulation [19]. To determine the estrus phase, the vaginal appearance is slightly swelling, less pink, has more striations, and is less moist [19]. This assessment was performed by experienced staff in the Department of Anatomy, Faculty of Veterinary Medicine, Airlangga University. After 4 weeks, blood samples were taken and then body weight was remeasured before euthanasia. Fecal microbial samples were obtained and followed by obtaining ovary tissue samples.

c. Bacterial Culture

The fecal content was taken aseptically and inoculated in peptone water overnight at 37 ° C. Sterile petri dishes was prepared which contained MacConkey Agar for Enterobacteriaceae. Each specimen was sub-cultured, grown, and eventually counted in CFU.

d. HOMA-IR and Testosterone Level Measurement

At the end of the treatment period, blood samples were obtained from the tail vein after fasting all rats for a period of 12 h. Fasting blood glucose was measured with a digital glucometer, while fasting insulin quantification was obtained using a rat insulin ELISA kit. To determine insulin resistance that occurs in rats, HOMA-IR was assessed by multiplying fasting plasma insulin (IU mg/mL) and fasting plasma glucose (mg/dL), divided by 405 [20]. The testosterone level was quantified using an ELISA kit from blood samples.

e. Hematoxylin and Eosin Staining

Once obtained, ovary samples were fixed in 10% neutral-buffered formaldehyde, treated with ethanol for dehydration, and then embedded in paraffin wax. Embedded paraffinized blocks were cut using a microtome (5 µm sections) and slices were placed on glass slides. The slides were held in staining racks, then incubated in three changes of xylene for paraffin removal. The slides were then hydrated with 100% ethanol three times, followed by 95% and 70% incubation in ethanol for 2 min each. Initially, hematoxylin dye was used for 3 min for slides and eosin Y was applied for 2 min before the slides were hydrated [21]. Slides were stained in hematoxylin and eosin to evaluate numbers of corpus luteum, antral and pre-ovulatory follicles, as well as the thickness of ovarian stroma.

f. Statistical Analysis

The Shapiro–Wilk test was applied to determine the data normality. When the data were not normally distributed, the chosen statistical test was the Mann–Whitney U test. Unpaired Student’s *t*-test was applied for data with a normal distribution. Pearson’s correlation was performed to determine the relationships among the variables. Data were expressed as mean ± SD or median (minimum–maximum value) with a statistically significant difference at *p* < 0.05.

## 3. Results

- HSCA did not lead to an overweight but reduced composition of the Enterobacteriaceae family in the gastrointestinal tract of female rats

**Figure 1:**
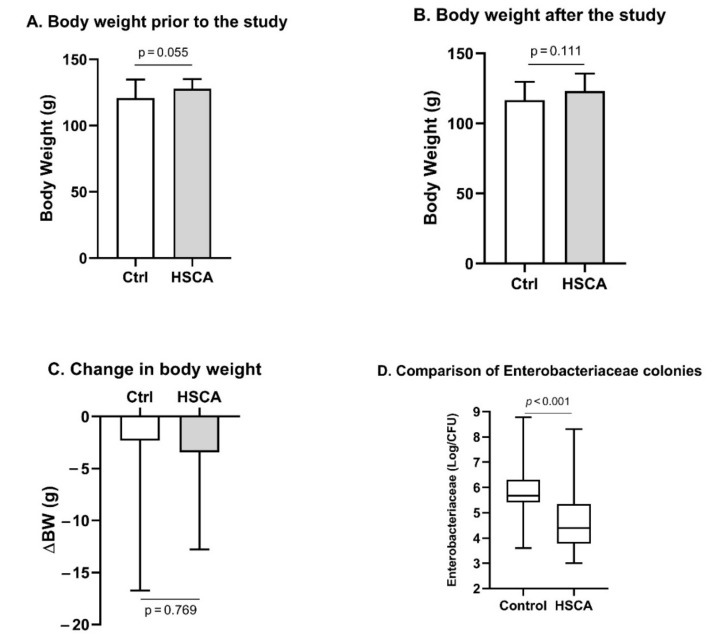
HSCA did not affect body weight but reduced *Enterobacteriaceae* composition in female rats. Rat body weight showed no difference between the two groups before and after the study (**A**,**B**). Body weight seemed reduced, but there was no significant difference between both groups (**C**). *Enterobacteriaceae* in HSCA rats was significantly lower than that in control rats (in CFU) (**D**). (**A**–**C**) Mean ± SD (Student’s *t*-test). (**D**) Median, with minimum and maximum values (Mann–Whitney U test). HSCA, high sucrose and cholic acid; ∆BW, the difference between body weight before and after the study; CFU, colony-forming unit.

To evaluate the effect of HSCA on the promotion of increased body weight, we measured body weight before and after food treatment periods. As presented in Figure 1A,B, HSCA had no effect on rat body weight, compared to rats treated with normal food. In fact, the treatment caused a reduction in body weight with an average of 3.45 g, from 127.75 to 123.2 g. Similarly, the control group expressed a body weight loss with an average of 2.31 g. However, as presented in Figure 1C, this weight loss was not significantly different between groups (*p* = 0.769).

A high level of cholic acid in the gut could be related to the alteration of the colonic microbiota [16,17]. In the present study, we obtained colonic microbial samples and assessed the colonies of the *Enterobacteriaceae* family from both groups, growing the bacteria on MacConkey Agar. *Enterobacteriaceae* are observed since many members of the family are considered pathobionts [22]. We performed the Shapiro–Wilk test for normality and found that the data were not normally distributed (Figure S1). Therefore, the Mann–Whitney U test was applied to assess the statistical difference. As presented in Figure 1D, numbers of *Enterobacteriaceae* in female rats treated with HSCA were 2.47 × 10^4^/CFU (min. 10^3^–max. 2 × 10^8^), significantly lower than in control rats, 4.75 × 10^5^/CFU (min. 4 × 10^3^–max. 6 × 10^8^), *p* < 0.001.

b. HSCA induced insulin resistance and hyperandrogenism in female Wistar rats

**Figure 2:**
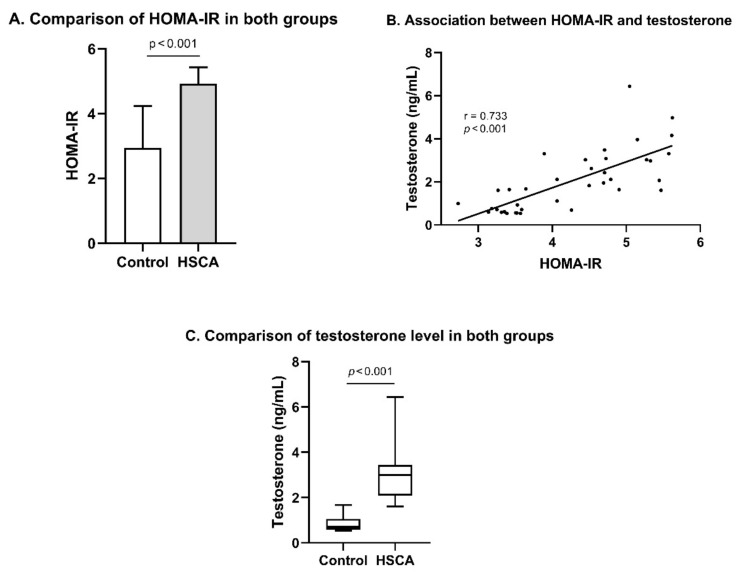
HSCA diet led to impaired insulin sensitivity and an enhanced testosterone level. HSCA diet increased HOMA-IR (multiplication of fasting insulin and blood glucose multiplication) (**A**). HOMA-IR, Homeostatic Model Assessment for Insulin Resistance. Data are presented as mean ± SD, statistical Student’s *t*-test. HOMA-IR was positively correlated with testosterone level (**B**), with a high level of testosterone presented in the HSCA-ingested rats, compared to that in control rats (**C**). Data are presented as median with minimum and maximum values, statistical Mann–Whitney U test.

To evaluate the effect of HSCA on impairing insulin sensitivity, blood samples were taken to measure fasting blood glucose and fasting insulin levels. This study found that HSCA-fed rats displayed significantly increased HOMA-IR, with a mean value of 4.92 ± 0.51, compared to control rats with HOMA-IR of 2.94 ± 1.3, *p* < 0.001 (Figure 2A). A well-established theory points out that insulin resistance, as well as an increased blood insulin level, is a vital element that alters insulin receptor-induced cellular signaling, thus upregulating the synthesis of androgenic hormones in theca cells [23,24]. In the present study, we demonstrated multiple Pearson’s correlation tests for all observed variables and found that there was a significant positive correlation between testosterone level and HOMA-IR (r = 0.733, *p* < 0.001; Figure S2 and Figure 2B).

To determine the level of testosterone, ELISA was used for obtaining the data from blood samples. After testing with the Shapiro–Wilk test, the measured testosterone level in this study was not normally distributed, so we did not employ Student’s *t*-test, but instead the Mann–Whitney U test for an appropriate statistical test (Figure S3). Elevated testosterone in HSCA rats was presented with an approximately 4.5-fold increase compared to the testosterone level in control rats (median 3 vs. 0.65 ng/mL, *p* < 0.001, Mann–Whitney test; Figure 2C).

c. HSCA exposure had no effect on ovulation, but induced impaired follicular maturation

**Figure 3:**
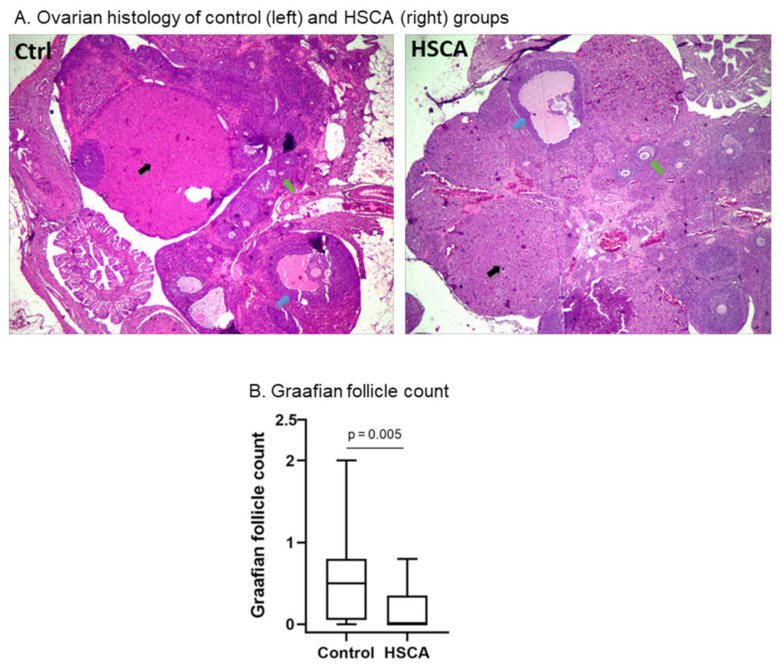
HSCA diet did not affect ovulation but deteriorated follicular maturation. Ovarian histology (magnification 40×) showed that corpus luteum (black arrow), tertiary (green arrow), and pre-ovulatory follicles (blue arrow) were presented in both groups (**A**). HSCA diet rats showed a reduced Graafian follicle count compared to control rats (**B**). Represented as median with minimum and maximum values.

To further explore the effect of the HSCA diet on ovarian function, several markers, including tertiary follicle, Graafian follicle, and corpus luteum, were counted and further analyzed. As presented in Figure 3A, all markers were detected in the ovarian histology. Interestingly, corpus luteum was shown in both HSCA and control rats, suggesting that the rats underwent ovulation (black arrow). We also counted numbers of corpus luteum and demonstrated no statistical differences between both groups (Figure S4).

Numbers of tertiary follicles between HSCA and control groups also showed no statistical difference (Figure 3A and Figure S4), while Figure 3A,B show that Graafian follicle counts in the HSCA group (median 0.00, min. 0.00–max. 0.80) were significantly lower than those in the control group (median 0.50, min. 0.00–max. 2.00), with *p* = 0.005 (Mann–Whitney U test, Figure S5). This suggests that HSCA could deteriorate the follicular maturation.

d. Numbers of tertiary follicles positively associated with corpus luteum countation

With the Pearson correlation test, numbers of tertiary follicles were positively correlated with corpus luteum count (*p* = 0.005, r = 0.437, Figures S2 and S4). As its name suggests, multiple small cysts are usually presented in the ovaries of PCOS patients [8]. However, in the present study, multiple cysts were not observed in both groups (Figure 3A).

## 4. Discussion

A high-sucrose diet (HSD) has been linked to increased body weight. A systematic review and meta-analysis showed that a reduction in dietary sugar intake was associated with weight loss, while an increase in sugar in the diet increased the risk of being overweight [25]. However, HSD does not induce overweight in rats [13,26]. Our findings showed that there was a slight reduction in body weight under HSCA treatment, but not significant, compared to rats with normal food (Figure 1A–C). A slight reduction in body weight can be associated with the administration of cholic acid [27]. Although HSD does not increase body weight, it causes glucose metabolic dysfunction, such as hyperglycemia and insulin resistance, in models of PCOS rats [13]. Approximately 70% of women with PCOS are phenotypically insulin-resistant [28]. In Figure 2A,C, increased insulin resistance as well as a high level of testosterone were observed in HSCA rats and may be associated with excessive sucrose and cholic acid ingestion [14,28]. However, Qi et al. showed that the administration of cholic acid in PCOS mice results in improved insulin sensitivity [16]. A future study with a design of three different treatments (HSD, CA, and HSCA) is required to confirm this finding.

*Enterobacteriaceae*, a family of Gram-negative bacteria, consist of commensals that provide benefits to the host and interact with other intestinal microbiotas. However, some members are considered pathobionts, such as Salmonella, Shigella, and Yersinia [22]. The family has been found increased in PCOS patients [17]. Before the study, we hypothesized that *Enterobacteriaceae* colonies would increase under HSCA treatment. De Oliveira Neves et al. showed an increase in *Enterobacteriaceae* groups and other pathobionts in Wistar rats, suggesting HSD-induced gut dysbiosis [29]. However, our findings showed the opposite (Figure 1D). Our study treated the animal with a combination of HSD and cholic acid, while de Oliveira Neves et al. fed rats with HSD [29]. Therefore, we considered that cholic acid might have a contrary effect to HSD. Nevertheless, this is only speculation. To confirm the effect of HSD and CA on *Enterobacteriaceae* and other gut microbiotas, future research is required with rigorous applications such as 16S rRNA gene sequencing, shotgun metagenomics, or microarray [29,30]. In the present study, the dysbiosis of the intestinal microbiota cannot be determined with a simple bacterial culture, but the result may reflect an alteration of the composition of the intestinal microbiota.

We hypothesized that HSCA treatments for four weeks would lead to abnormal folliculogenesis. However, the HSCA diet did not influence all the steps of folliculogenesis, but only affected follicular maturation. The reason for this event remains unknown, but we considered that an interaction of complex factors, such as insulin resistance and high levels of testosterone, induces ovarian malfunctions [13,31]. Insulin resistance could affect folliculogenesis by increasing the biosynthesis of testosterone in theca cells [2,31]. However, the researchers argued that insulin resistance in ovaries generates a change in glucose metabolism but remains unchanged in steroidogenesis and cell proliferation [24]. The exact mechanism of insulin to preserve its function in steroidogenesis remains elusive, but insulin likely acts through IGF-1 or hybrid insulin/IGF-1 receptors [32].

**Figure 4:**
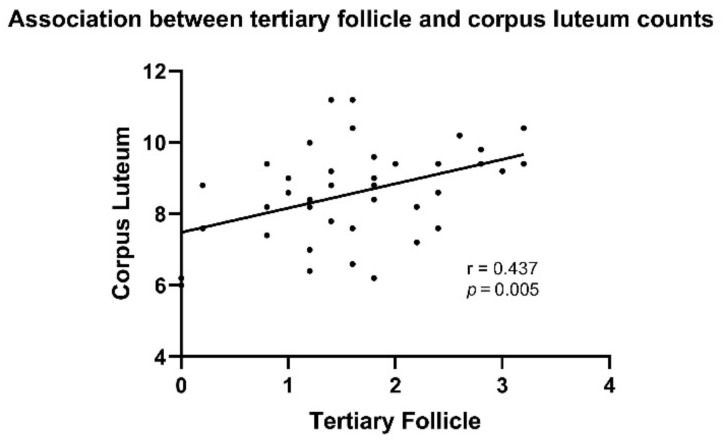
Numbers of tertiary follicles positively correlated with corpus luteum count.

In addition, this study also did not find the presence of ovarian cysts, while de Melo [13] have shown increased cystic follicles in HSD-treated compared to control rats. Although ovulation is not affected in HSD rats, other PCOS phenotypes are present, such as a greater number of ovarian cysts, an earlier vaginal opening, and an increase in the atretic antral follicles [13]. They designed the study with 14 weeks of treatment, whereas our study was a shorter period (4 weeks). We speculate that our findings, as shown in Figure 3A, are an early sign of folliculogenesis deterioration, as longer periods of HSCA treatment could result in more prominent PCOS phenotypes. In addition, we found that there is a correlation between tertiary follicles and corpus luteum (Figure 4). Deterioration in the follicular phase could be related to abnormality in the luteal phase [33]. However, in the present study, corpus luteum counts between two groups did not show significant differences (Figure S4). The luteal phase may also be affected during longer exposure to HSCA. More studies are required to confirm our speculation by evaluating the ovarian every 4 weeks, with 16 weeks of treatment periods.

## 5. Conclusions

The data presented in this study suggested that HSCA causes rats to develop insulin resistance and high testosterone levels, which in turn abrogate ovarian follicular maturity in PCOS. The mechanism may involve the alteration of the gut microbiota, marked by decreased composition of Enterobacteriaceae. More studies are required to profile the intestinal microbiota to determine intestinal dysbiosis and observe more PCOS phenotypes with a longer study duration (14 weeks or more) in animals treated with HSCA.

## Data Availability

Not applicable.

## Supplementary Materials

The following supporting information can be downloaded at: https://www.mdpi.com/article/10.3390/pathophysiology29030026/s1, Figure S1: Shapiro–Wilk test for the data normality of Enterobacteriaceae composition. Both control and HSCA groups showed *p* < 0.05. Figure S2: Multiple correlation analysis among measured variables. The data indicate the Pearson coefficient. ** *p* < 0.01, *** *p* < 0.001. Figure S3: Shapiro–Wilk test for the data normality of testosterone levels. Both control and HSCA groups showed *p* < 0.05. Figure S4: Counts of corpus luteum and tertiary follicles. Data are represented as mean ± SD. Figure S5: Shapiro–Wilk test for the data normality of Graafian follicle count. Both control and HSCA groups showed *p* < 0.05.

## References

[1] Williams T., Mortada R., Porter S. (2016). Diagnosis and Treatment of Polycystic Ovary Syndrome. Am. Fam. Physician.

[2] Franks S., Henry H.L., Norman A.W. (2003). Polycystic Ovary Syndrome. Encyclopedia of Hormones.

[3] Tabassum F., Jyoti C., Sinha H.H., Dhar K., Akhtar M.S. (2021). Impact of polycystic ovary syndrome on quality of life of women in correlation to age, basal metabolic index, education and marriage. PLoS ONE.

[4] Wolf W.M., Wattick R.A., Kinkade O.N., Olfert M.D. (2018). Geographical Prevalence of Polycystic Ovary Syndrome as Determined by Region and Race/Ethnicity. Int. J. Environ. Res. Public Health.

[5] Deswal R., Narwal V., Dang A., Pundir C.S. (2020). The Prevalence of Polycystic Ovary Syndrome: A Brief Systematic Review. J. Hum. Reprod. Sci..

[6] Liu J., Wu Q., Hao Y., Jiao M., Wang X., Jiang S., Han L. (2021). Measuring the global disease burden of polycystic ovary syndrome in 194 countries: Global Burden of Disease Study 2017. Hum. Reprod..

[7] Belenkaia L.V., Lazareva L.M., Walker W., Lizneva D.V., Suturina L.V. (2019). Criteria, phenotypes and prevalence of polycystic ovary syndrome. Minerva Ginecol..

[8] Giampaolino P., Foreste V., Di Filippo C., Gallo A., Mercorio A., Serafino P., Improda F., Verrazzo P., Zara G., Buonfantino C. (2021). Microbiome and PCOS: State-of-Art and Future Aspects. Int. J. Mol. Sci..

[9] Rosenfield R.L., Ehrmann D.A. (2016). The Pathogenesis of Polycystic Ovary Syndrome (PCOS): The Hypothesis of PCOS as Functional Ovarian Hyperandrogenism Revisited. Endocr. Rev..

[10] Merkin S.S., Phy J.L., Sites C.K., Yang D. (2016). Environmental determinants of polycystic ovary syndrome. Fertil. Steril..

[11] Barrea L., Arnone A., Annunziata G., Muscogiuri G., Laudisio D., Salzano C., Pugliese G., Colao A., Savastano S. (2019). Adherence to the Mediterranean Diet, Dietary Patterns and Body Composition in Women with Polycystic Ovary Syndrome (PCOS). Nutrients.

[12] Leeming E.R., Johnson A.J., Spector T.D., Le Roy C.I. (2019). Effect of Diet on the Gut Microbiota: Rethinking Intervention Duration. Nutrients.

[13] De Melo G.B., Soares J.F., Costa T.C.L., Benevides R.O.A., Vale C.C., Paes A.M.D.A., Gaspar R.S. (2021). Early Exposure to High-Sucrose Diet Leads to Deteriorated Ovarian Health. Front. Endocrinol..

[14] Yang X., Wu R., Qi D., Fu L., Song T., Wang Y., Bian Y., Shi Y. (2021). Profile of Bile Acid Metabolomics in the Follicular Fluid of PCOS Patients. Metabolites.

[15] Zhang B., Shen S., Gu T., Hong T., Liu J., Sun J., Wang H., Bi Y., Zhu D. (2019). Increased circulating conjugated primary bile acids are associated with hyperandrogenism in women with polycystic ovary syndrome. J. Steroid Biochem. Mol. Biol..

[16] Qi X., Yun C., Sun L., Xia J., Wu Q., Wang Y., Wang L., Zhang Y., Liang X., Gonzalez F.J. (2019). Gut microbiota–bile acid–interleukin-22 axis orchestrates polycystic ovary syndrome. Nat. Med..

[17] Zhu X., Li Y., Jiang Y., Zhang J., Duan R., Liu L., Liu C., Xu X., Yu L., Wang Q. (2021). Prediction of Gut Microbial Community Structure and Function in Polycystic Ovary Syndrome with High Low-Density Lipoprotein Cholesterol. Front. Cell. Infect. Microbiol..

[18] Marcondes F.K., Bianchi F.J., Tanno A.P. (2002). Determination of the estrous cycle phases of rats: Some helpful considerations. Braz. J. Biol..

[19] Ajayi A.F., Akhigbe R.E. (2020). Staging of the estrous cycle and induction of estrus in experimental rodents: An update. Fertil. Res. Pr..

[20] Ghiasi R., Soufi F.G., Somi M.H., Mohaddes G., Bavil F.M., Naderi R., Alipour M.R. (2015). Swim Training Improves HOMA-IR in Type 2 Diabetes Induced by High Fat Diet and Low Dose of Streptozotocin in Male Rats. Adv. Pharm. Bull..

[21] Cardiff R.D., Miller C.H., Munn R.J. (2014). Manual hematoxylin and eosin staining of mouse tissue sections. Cold Spring Harb. Protoc..

[22] Kang E., Crouse A., Chevallier L., Pontier S.M., Alzahrani A., Silué N., Campbell-Valois F.-X., Montagutelli X., Gruenheid S., Malo D. (2018). Enterobacteria and host resistance to infection. Mamm. Genome.

[23] Dunaif A., Segal K.R., Shelley D.R., Green G., Dobrjansky A., Licholai T. (1992). Evidence for Distinctive and Intrinsic Defects in Insulin Action in Polycystic Ovary Syndrome. Diabetes.

[24] Dunaif A. (1997). Insulin Resistance and the Polycystic Ovary Syndrome: Mechanism and Implications for Pathogenesis. Endocr. Rev..

[25] Te Morenga L., Mallard S., Mann J. (2013). Dietary sugars and body weight: Systematic review and meta-analyses of randomised controlled trials and cohort studies. BMJ.

[26] Kanazawa M., Xue C.Y., Kageyama H., Suzuki E., Ito R., Namba Y., Osaka T., Kimura S., Inoue S. (2003). Effects of a high-sucrose diet on body weight, plasma triglycerides, and stress tolerance. Nutr. Rev..

[27] Do H.J., Lee Y.S., Ha M.J., Cho Y., Yi H., Hwang Y.-J., Hwang G.-S., Shin M.-J. (2016). Beneficial effects of voglibose administration on body weight and lipid metabolism via gastrointestinal bile acid modification. Endocr. J..

[28] Moghetti P. (2016). Insulin Resistance and Polycystic Ovary Syndrome. Curr. Pharm. Des..

[29] Neves V.G.d.O., de Oliveira D.T., Oliveira D.C., Perucci L.O., dos Santos T.A.P., Fernandes I.d.C., de Sousa G.G., Barboza N.R., Guerra-Sá R. (2020). High-sugar diet intake, physical activity, and gut microbiota crosstalk: Implications for obesity in rats. Food Sci. Nutr..

[30] Wei S., Bahl M.I., Baunwall S.M.D., Hvas C.L., Licht T.R. (2021). Determining Gut Microbial Dysbiosis: A Review of Applied Indexes for Assessment of Intestinal Microbiota Imbalances. Appl. Environ. Microbiol..

[31] Li A., Zhang L., Jiang J., Yang N., Liu Y., Cai L., Cui Y., Diao F., Han X., Liu J. (2017). Follicular hyperandrogenism and insulin resistance in polycystic ovary syndrome patients with normal circulating testosterone levels. J. Biomed. Res..

[32] Poretsky L. (1991). On the Paradox of Insulin-Induced Hyperandrogenism in Insulin-Resistant States. Endocr. Rev..

[33] Abdulla S.H., Bouchard T.P., Leiva R.A., Boyle P., Iwaz J., Ecochard R. (2018). Hormonal Predictors of Abnormal Luteal Phases in Normally Cycling Women. Front. Public Health.

