# Supplementary material for: High Sucrose and Cholic Acid Diet Triggers PCOS-like Phenotype and Reduces Enterobacteriaceae Colonies in Female Wistar Rats
Source: Pathophysiology. 2022 Jul 8;29(3):344–53. doi: 10.3390/pathophysiology29030026 (PMC9326763; doi:10.3390/pathophysiology29030026)
Supplement: Supplementary file 1 [file pathophysiology-29-00026-s001.zip › pathophysiology-1743973-supplementary.pdf]

Supplementary Figures

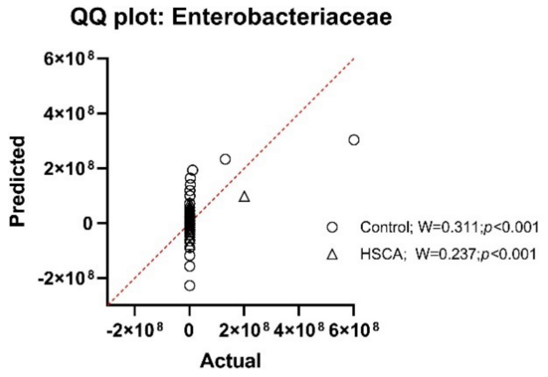

Supplementary Figure S1. Shapiro-Wilk test for the data normality of Enterobacteriaceae composition. Both control and HSCA groups showed $p < 0.05$.

Pearson Coefficient	Enterobacteriaceae	HOMA-IR	Testosterone	Tertiary Follicle	Graafian follicle	Corpus Luteum
Enterobacteriaceae	1	0.071	-0.156	0.056	-0.047	0.062
HOMA-IR	0.071	1	0.733***	-0.054	-0.236	-0.081
Testosterone	-0.156	0.733***	1	-0.232	-0.216	0.008
Tertiary Follicle	0.056	-0.054	-0.232	1	-0.002	0.437**
Graafian follicle	-0.047	-0.236	-0.216	-0.002	1	-0.133
Corpus Luteum	0.062	-0.081	0.008	0.437**	-0.133	1

Supplementary Figure S2. Multiple correlation analysis among measured variables. The data indicated Pearson coefficient. ** $p<0.01$, *** $p<0.001$.

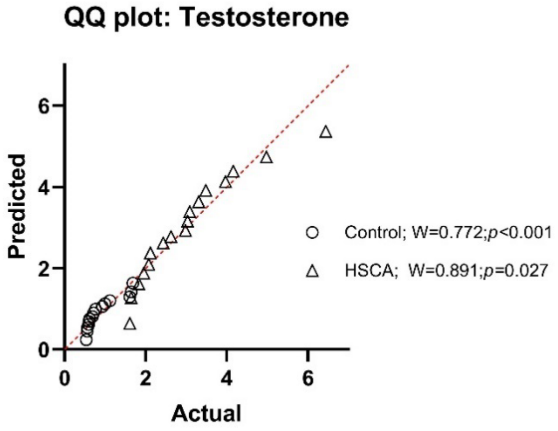

Supplementary Figure S3. Shapiro-Wilk test for the data normality of testosterone level. Both control and HSCA groups showed $p<0.05$.

	Control	HSCA	*p*-value
Corpus luteum	8.97 ± 1.61	8.46 ± 1.03	0.642
Tertiary follicle	1.68 ± 0.83	1.48 ± 0.89	0.467

Supplementary Figure S4. Counts of corpus luteum and tertiary follicle. Data represented as Mean ± SD**.**

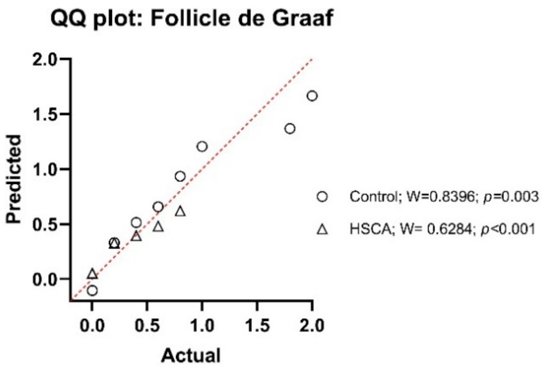

Supplementary Figure S5. Shapiro-Wilk test for the data normality of Graafian follicle count. Both control and HSCA groups showed $p<0.05$.
